# Role of Calcitonin Gene-Related Peptide in Bone Repair after Cyclic Fatigue Loading

**DOI:** 10.1371/journal.pone.0020386

**Published:** 2011-06-09

**Authors:** Susannah J. Sample, Zhengling Hao, Aliya P. Wilson, Peter Muir

**Affiliations:** Comparative Orthopaedic Research Laboratory, School of Veterinary Medicine, University of Wisconsin-Madison, Madison, Wisconsin, United States of America; University of Pittsburgh, United States of America

## Abstract

**Background:**

Calcitonin gene related peptide (CGRP) is a neuropeptide that is abundant in the sensory neurons which innervate bone. The effects of CGRP on isolated bone cells have been widely studied, and CGRP is currently considered to be an osteoanabolic peptide that has effects on both osteoclasts and osteoblasts. However, relatively little is known about the physiological role of CGRP *in-vivo* in the skeletal responses to bone loading, particularly fatigue loading.

**Methodology/Principal Findings:**

We used the rat ulna end-loading model to induce fatigue damage in the ulna unilaterally during cyclic loading. We postulated that CGRP would influence skeletal responses to cyclic fatigue loading. Rats were fatigue loaded and groups of rats were infused systemically with 0.9% saline, CGRP, or the receptor antagonist, CGRP_8–37_, for a 10 day study period. Ten days after fatigue loading, bone and serum CGRP concentrations, serum tartrate-resistant acid phosphatase 5b (TRAP5b) concentrations, and fatigue-induced skeletal responses were quantified. We found that cyclic fatigue loading led to increased CGRP concentrations in both loaded and contralateral ulnae. Administration of CGRP_8–37_ was associated with increased targeted remodeling in the fatigue-loaded ulna. Administration of CGRP or CGRP_8–37_ both increased reparative bone formation over the study period. Plasma concentration of TRAP5b was not significantly influenced by either CGRP or CGRP_8–37_ administration.

**Conclusions:**

CGRP signaling modulates targeted remodeling of microdamage and reparative new bone formation after bone fatigue, and may be part of a neuronal signaling pathway which has regulatory effects on load-induced repair responses within the skeleton.

## Introduction

The failure of repair responses to protect the skeleton from fracture is an important problem, but the physiological pathways that regulate skeletal responses to loading are not fully understood. Fractures resulting from osteoporosis were estimated to cost the U.S.A. $17 billion in 2005, a number that is expected to rise nearly 50% by 2025 [1]. In addition to osteoporotic fracture, stress fractures are also common in military recruits and other human, canine and equine athletes, where bone fatigue can overwhelm repair mechanisms, with associated accumulation of fatigue damage within bone and development of a stress fracture [2]–[4].

Functional adaptation of the skeleton is thought to consist primarily of two processes, modeling and remodeling [5]. Bone modeling changes the spatial distribution of bone, while remodeling is a process of bone removal and replacement [5]. In situations where fatigue damage is present within bone, repair responses include targeted remodeling of microdamage [6] and woven bone formation on adjacent bone surfaces. Load-induced skeletal responses are thought to be locally regulated by bone cells [7], [8]. However, recent work also suggests that the sensory innervation of bone may have regulatory effects on skeletal responses to bone loading [9], [10].

The nervous system plays a role in the regulation of skeletal metabolism [11]. The sensory innervation of bone also has an important role in nociception and development of bone pain [12]. However, little work to date has addressed whether or not the innervation of bone has a functional role in the physiological responses of bone to loading. Periosteum, endosteum, and bone tissue are all innervated by nerve fibers [13]–[15]. This innervation exhibits plasticity in response to mechanical loading, in that a single loading event results in persistent changes in neuropeptide concentrations in both loaded and distant long bones, as well as changes in the neural circuits between limbs [9], [10]. Of the three compartments of long bones, the periosteum has a particularly dense innervation, which is arranged in a net-like meshwork optimized for the detection of mechanical distortion [12]. This innervation is primarily peptidergic, and contains both sensory and sympathetic fibers [12], [16], [17]. Individual bone cells are directly connected to the nervous system via unmyelinated sensory neurons [18]. Bone cells express a range of functional neurotransmitter receptors and transporters, including those for calcitonin gene related peptide (CGRP) [19]–[21].

The calcitonin family of peptides has been extensively studied in bone over the past few years because of their effects on bone cells and potential as future drug targets. CGRP has pleiotropic effects on bone cells; both osteoclasts and osteoblasts have functional receptors for CGRP [20]–[22]. Physiological actions of CGRP are mediated through a family of type II G-protein coupled receptors, the most important of which is the CGRP_1_ receptor [20], [23], [24]. In vitro, CGRP inhibits maturation of osteoclasts [25] and bone resorption [26], and is anabolic to osteoblasts by stimulation of canonical Wnt signaling and by inhibition of osteoblast apoptosis [21], [27]. CGRP has two isoforms, CGRP-α and CGRP-β. In the rat, CGRP-α and CGRP-β differ by only one amino acid, despite being derived from separate genes, *Calca* and *Calcb*, respectively [28]. CGRP-α increases the proliferation rate of osteoblasts [27], [29], prevents bone loss when delivered systemically to ovariectomized rats [30], and increases bone mass in transgenic mice with an osteoblast-specific promoter that over-expresses CGRP-α [31]. CGRP-α knockout mice are osteopenic [32]. Collectively, these findings suggest that CGRP-α is a peptide with osteoanabolic activity *in-vivo*. Unlike CGRP-α, CGRP-β is not considered osteogenic [33].

In the present study, our goal was to determine whether bone CGRP concentrations were modulated by cyclic fatigue loading *in vivo*. An increase would be expected with an osteoanabolic effect in a bone formation model, although earlier work suggests that short periods of loading without fatigue are associated with reduced CGRP concentrations in bone [9]. Using the fatigue loading model, we also sought to determine whether manipulation of systemic CGRP signaling *in vivo* would limit osteoclast activation for remodeling and increase reparative osteogenesis.

## Materials and Methods

### Animals

A homogeneous group of 68 actively growing male Sprague-Dawley rats (body weight 292–305 g, aged 67±14 days) was used for the study. Rats were provided with food and water ad libitum. All procedures were performed in strict accordance with the recommendations in the Guide for the Care and Use of Laboratory Animals of the National Institutes of Health and the American Veterinary Medical Association and with approval from the Animal Care Committee of the University of Wisconsin-Madison. Ulna loading was performed under isoflurane anesthesia with butorphanol analgesia. Humane euthanasia was performed under isoflurane anesthesia at the end of the experimental period, using an intracardiac injection of pentobarbitone.

### Experimental design

To determine the effect of unilateral ulna fatigue loading on CGRP peptide concentrations in the thoracic limb long bones, 12 rats were fatigue loaded until 40% loss of stiffness was attained, using an initial peak strain of −3,000 µε (Fatigue group). An additional 24 rats were used as controls: 12 rats were sham controls (Sham group), and thus given the same treatment regimen as rats in the treatment groups, but without being subjected to any mechanical loading; 12 rats served as baseline controls (Baseline group). Rats were euthanatized 10 days after loading or sham loading.

To determine how manipulation of systemic CGRP signaling might influence skeletal repair responses to fatigue loading, 32 rats were assigned one of 3 treatment groups. The right ulna of all rats was fatigue loaded until 40% of stiffness was attained, using an initial peak strain of −3,000 µε. Immediately after loading, an osmotic pump (Alzet Corporation, Cupertino, CA) was implanted subcutaneously dorsally between the scapulae to provide a continuous infusion of either saline, CGRP, or the CGRP_1_ receptor antagonist CGRP_8–37_ [23], [34], for the duration of the study. The 8 rats assigned to the Saline group received 40 µl/kg/day 0.9% saline. The 12 rats assigned to the CGRP group received 100 µg/kg/day of CGRP [35] and the 12 rats assigned to the CGRP_8–37_ group received 100 µg/kg/day of CGRP_8–37_ [36]. All rats were additionally given an intraperitoneal injection of calcein (7 mg/kg) immediately after fatigue loading and again 7 days later to label load-induced bone formation. Rats were euthanatized 10 days after loading.

### In-vivo ulnar fatigue loading

**Figure 1:**
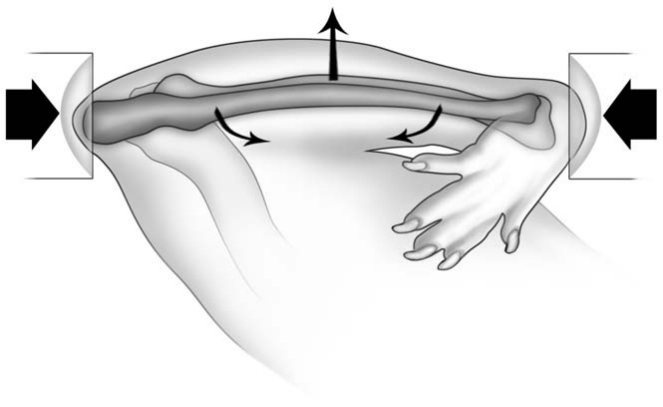
Schematic diagram of the rat ulna loading model. The antebrachium was placed horizontally in loading cups attached to a materials testing machine. The medio-lateral diaphyseal curvature of the rat ulna is accentuated through axial compression, most of which is translated into a bending moment, which is greatest at ∼60% the total bone length measured from the proximal end of the ulna [37]. Ulnae underwent cyclic fatigue loading, initiated at −3,000 µε, with incremental increases in load until fatigue was initiated. Loading was then terminated when 40% loss of stiffness was attained. Reproduced from [43] with permission from John Wiley & Sons.

*In-vivo* fatigue loading of the right ulna was performed under isoflurane-induced general anesthesia. The right antebrachium of each rat was placed horizontally between two loading cups, which were fixed to the loading platen and actuator of a materials testing machine (Model 8800 DynaMight; Instron, Canton, MA, USA) with a 250N load cell (Honeywell Sensotec, Canton, MA, USA). The right ulna then underwent cyclic loading by means of axial compression, which accentuates the pre-existing mediolateral curvature of the diaphysis of the rat ulna, translating most of the axial force into a bending moment (Fig. 1). Cyclic fatigue loading was performed at 4 Hz, and was initiated at −16N. To induce fatigue, the load applied to the ulna was incrementally increased until fatigue was initiated, as indicated by increasing displacement amplitude from a stable baseline. Loading was then terminated when 40% loss of stiffness was attained.

### Quantification of plasma and bone CGRP and plasma TRAP5b

At the time of euthanasia, plasma blood samples were collected and left and right ulnae were dissected with surrounding tissue; all samples were stored at −80°C until processing. Bones were placed in liquid nitrogen and pulverized using a ball-mill grinder (Mikro-Dismembrator S; Sartorius Stedim Biotech, Aubagne Cedex, France). After homogenization, the samples were centrifuged at 3,000 *g* for 15 min. The supernatants were freeze-dried and dissolved in ELISA buffer. Plasma and bone CGRP concentrations were determined using a rat-specific ELISA assay (Cayman Chemical, Ann Arbor, MI, USA). Total protein concentrations were also determined for both plasma and bone (BCA Protein assay, Thermo Scientific, Rockford, IL, USA). Plasma TRAP5b concentrations were determined using an ELISA kit validated for the rat (Immunodiagnostic Systems Ltd, Fountain Hills, AZ, USA). For each sample, CGRP and TRAP5b concentrations were normalized to the total protein concentration.

### Bone Histomorphometry

The pairs of ulnae and humeri were dissected along with surrounding tissue. Fluorochrome labeled bones were dehydrated in a graded series of ethanol (70%, 100%), bulk stained with Villanueva's solution for 3 days, and then embedded in methylmethacrylate. Transverse calcified sections, 125 µm thick, were made and mounted on standard microscope slides. Ulnae were sectioned at 60% of total bone length measured from the proximal end, where it has been shown maximal adaptation occurs with this model [37]. Humeri were sectioned at the mid-diaphysis (50% of total bone length). Confocal microscopy (MRC-1024 Laser Scanning Confocal Microscope; Bio-Rad, Hercules, CA, USA) was used to collect fluorescent images of each bone section. Periosteal and endosteal labeled bone areas (Ps.L.B.Ar and Es.L.B.Ar, %) were determined using a standard method [38], and total cortical bone area (Tt.L.B.Ar, %) was also determined by thresholding the image and pixel-counting (Image J; NIH) [9]. All measurements were made by a single observer (SJS). Data were normalized to the original cortical area to account for minor variations in rat size. Using the same sections, resorption space number density and microcrack surface density (Rs.Sp.Dn, #/mm^2^; Cr.S.Dn, µm/mm^2^, respectively) were also determined in the left and right ulnae by assessment of Villanueva staining using bright-field microscopy.

### Statistical Analysis

The Kolmogorov-Smirnov test was used to confirm that data were normally distributed. Limbs were treated as separate experiments. Group differences in normalized bone CGRP concentrations, normalized plasma CGRP and TRAP5b concentrations, and labeled adaptive bone formation were determined using a one-way ANOVA with a Dunnett post-hoc test; the Baseline group served as the control. Planned comparisons were used to determine differences between the CGRP and CGRP_8–37_ groups. The Kruskal-Wallis ANOVA test and the Mann-Whitney U test were use to analyze Rs.Sp.Dn and Cr.S.Dn. Results were considered significant at *p*<0.05. Data are reported as mean ± standard error of the mean or median and range for non-parametric data.

## Results

The number of cycles required to reach 47±15% loss of stiffness in fatigue-loaded ulnae was 3,738±2,472, with a final peak load of −24.0±4.0N. A minimally displaced intracortical stress fracture was noted in 31 of the 32 fatigue-loaded ulnae evaluated for microdamage; as expected, no intracortical fatigue damage was seen in the contralateral ulnae.

### Infusion of CGRP elevated plasma CGRP concentrations but did not influence plasma TRAP5b

**Figure 2:**
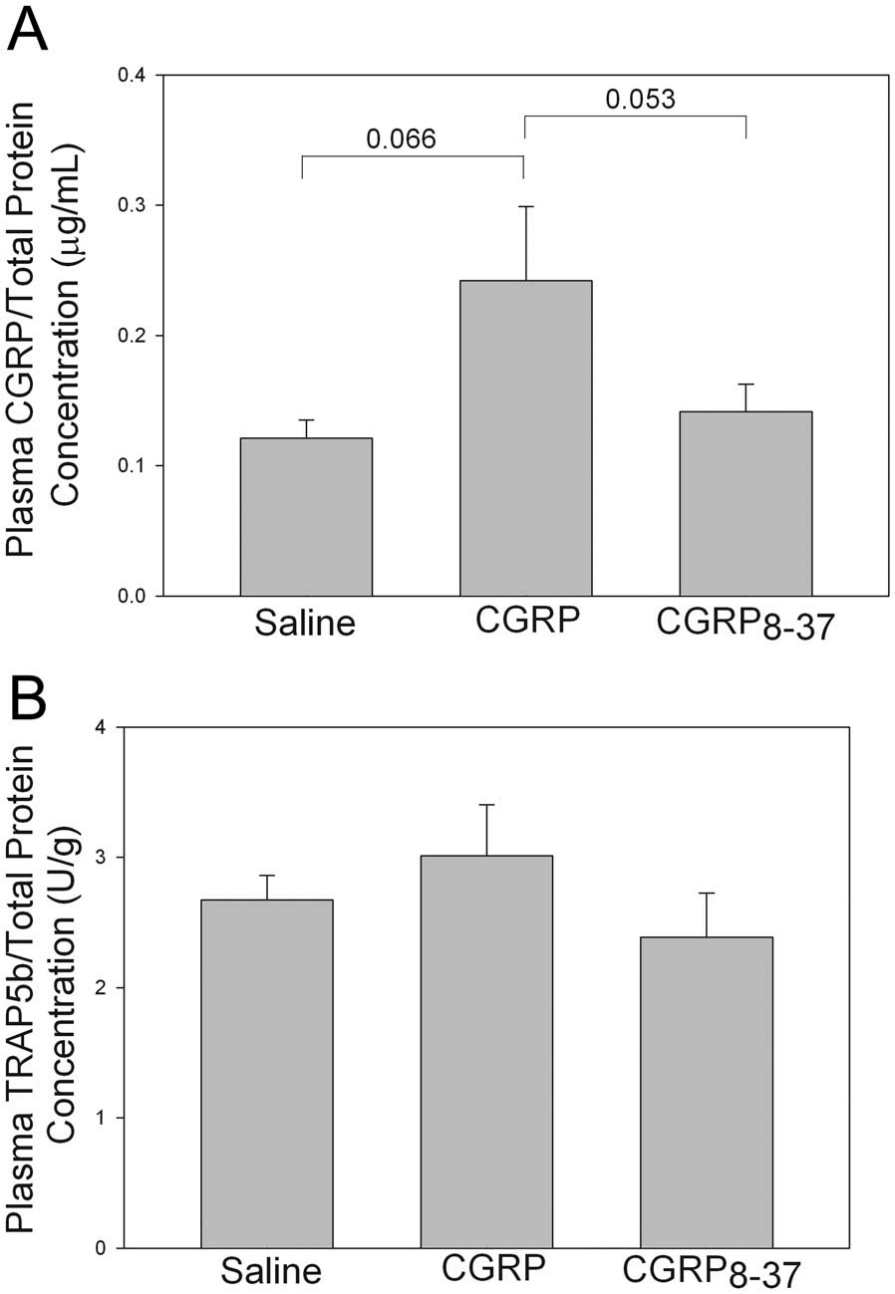
CGRP or CGRP_8–37_ administration did not influence plasma TRAP5b in vivo. Plasma concentrations of CGRP and TRAP5b, normalized to plasma total protein, 10 days after fatigue loading. (**A**) Rats in the CGRP group had higher plasma CGRP concentrations when compared to rats in the Saline and CGRP_8–37_ groups. No differences were seen between the Saline group and the CGRP_8–37_ group. (**B**) Administration of CGRP or CGRP_8–37_ did not have an effect on plasma TRAP5b levels. Error bars represent standard error of the mean. Saline group, n = 8; CGRP group n = 12; CGRP_8–37_ group, n = 12.

Plasma CGRP concentrations were measured to verify functional infusion of CGRP in the CGRP treatment group. Plasma CGRP was elevated in the CGRP group compared with the CGRP_8–37_ group (*p* = 0.05), but not the Saline group (*p*<0.07) (Fig. 2A). Plasma TRAP5b concentrations were not altered as a result of CGRP or CGRP_8–37_ administration (*p* = 0.41) (Fig. 2B).

### Cyclic fatigue loading increased bone CGRP concentrations in loaded and contralateral ulnae

**Figure 3:**
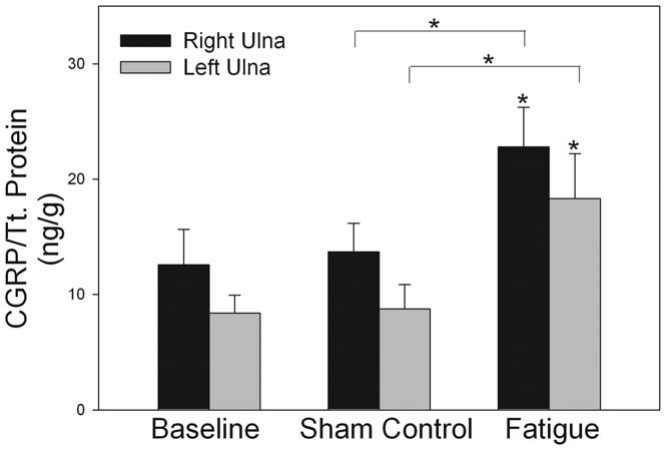
Bone CGRP is increased by mechanical loading. Cyclic fatigue loading of the right ulna resulted in increased CGRP concentrations in both the fatigue-loaded (right) ulna and the contralateral (left) ulna, when compared to the Baseline group. No differences in CGRP concentrations were seen between the Sham group and the Baseline group. The Fatigue group also had increased CGRP concentrations compared to the Sham group. * −*p*<0.05 versus the relevant baseline control bone. Error bars represent standard error of the mean. Baseline group n = 12; Sham group n = 12; Fatigue group n = 12.

To determine the effects of cyclic fatigue loading on bone CGRP concentrations, we quantified CGRP in both the loaded and contralateral ulnae of fatigue-loaded and control rats. The concentration of CGRP was increased in both the loaded right ulna (*p*<0.05) and the contralateral left ulna (*p*<0.05) of rats in the Fatigue group, when compared to the Baseline group (Fig. 3). No differences were seen between the Baseline group and the Sham group (*p* = 0.95). Additionally, no differences in humeral CGRP concentrations were seen between groups.

### Systemic administration of CGRP and CGRP_8–37_ altered bone remodeling in response to unilateral cyclic fatigue loading

**Figure 4:**
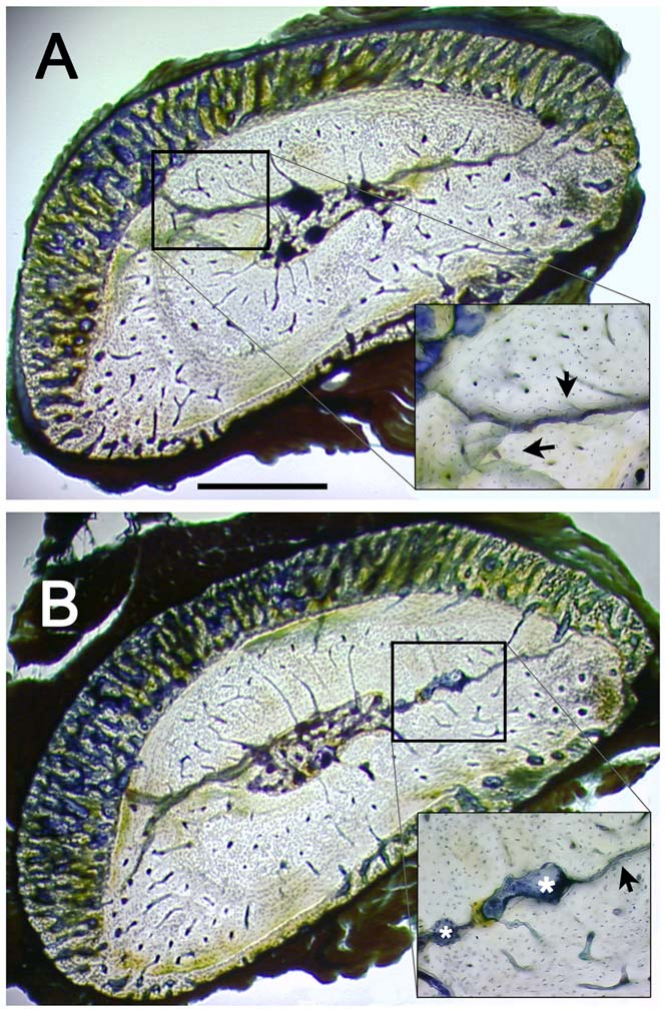
Targeted remodeling of bone microdamage. Photomicrographs of calcified transverse sections of ulna at 60% of bone length, from proximal to distal [37]. Fatigue loading induced microcrack formation and targeted remodeling. (**A**) Branching microcracks can be appreciated histologically in fatigue-loaded bones. (**B**) Targeted remodeling resulted in resorption space formation around the areas of microcracking. Bones were bulk-stained with Villanueva bone stain. Black arrows indicate fatigue damage; white asterisks are labeling resorption spaces. Bar = 0.5 mm.

**Figure 5:**
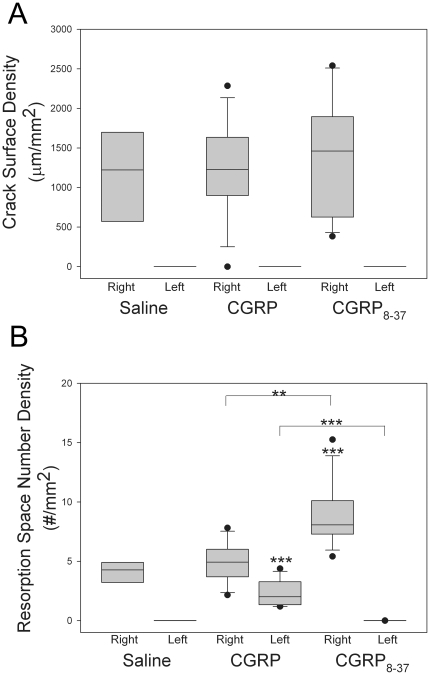
Remodeling of fatigue loaded bone was increased with administration of CGRP_8–37_. Treatment with either saline, CGRP or CGRP_8–37_ for 10 days after cyclic fatigue loading of the right ulna did not affect crack surface density (Cr.S.Dn), but resulted in altered resorption space density (Rs.Sp.Dn). (**A**) Cr.S.Dn was similar between groups. (**B**) Rs.Sp.Dn in the right ulna was increased in the CGRP_8–37_ group compared to the CGRP group and the Saline group. In the left ulna, resorption space number density was increased in the CGRP group, compared to the Saline group and the CGRP_8–37_ group. ** −*p*<0.01; *** −*p*<0.001; versus the relevant saline control bone. Error bars represent range. Saline group n = 8; CGRP group n = 12; CGRP_8–37_ group n = 12.

To quantify fatigue damage and associated osteoclast activation in rats treated with CGRP or CGRP_8–37_, we measured Cr.S.Dn and Rs.Sp.Dn in both fatigue-loaded right ulnae and the contralateral left ulnae (Fig. 4). No differences in Cr.S.Dn were seen between groups (Fig. 5A). Rats in the CGRP_8–37_ group had increased Rs.Sp.Dn in their right ulna 10 days after fatigue loading, compared to the CGRP group (*p*<0.001), and the Saline group (*p*<0.01) (Fig. 5B). In the contralateral left ulna, Rs.Sp.Dn was increased to a small extent in the CGRP group, compared to both the Saline group (*p*<0.001) and CGRP_8–37_ group (*p*<0.001).

### Systemic administration of both CGRP and CGRP_8–37_ increased reparative bone formation after unilateral cyclic fatigue loading

**Figure 6:**
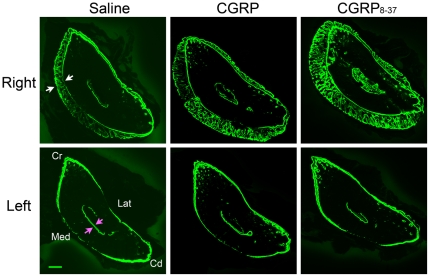
Reparative bone formation induced by fatigue loading was increased after treatment with CGRP or CGRP_8–37_. Confocal photomicrographs of calcified transverse sections of ulna at 60% of bone length, from proximal to distal [37]. Administration of either CGRP or CGRP_8–37_ for 10 days after cyclic fatigue loading of the right ulna increased reparative bone formation in the loaded ulna compared with saline-treated rats. Endosteal bone formation was particularly evident after CGRP_8–37_ treatment. Rats treated with CGRP_8–37_ also had greater bone formation in the contralateral (left) ulna, which was not loaded, when compared to the left ulna of the saline-treated rats. New bone formation was double labeled with calcein. White arrows indicate periosteal new woven bone formation; pink arrows indicate endosteal new bone. Bar = 250 µm. Cr, cranial; Cd, caudal; Med, medial; Lat, lateral. Saline group, n = 8; CGRP group n = 12; CGRP_8–37_ group, n = 12.

**Figure 7:**
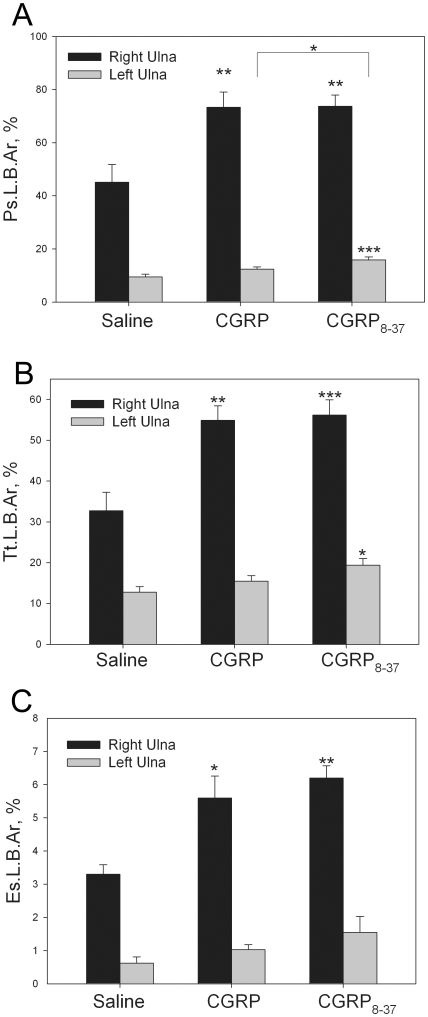
Calcein-labeled new bone formation in response to unilateral cyclic fatigue loading of the right ulna in rats. Treatment with either CGRP or CGRP_8–37_ for 10 days after fatigue loading the right ulna resulted in increased reparative bone formation in the loaded ulna in both treatment groups; treatment with CGRP_8–37_ also significantly influenced labeled bone formation in the contralateral ulna, when compared to saline treated rats. (**A**) Periosteal labeled bone area (Ps.L.B.Ar) was increased in the right ulnae of both treatment groups, and the left ulna of the CGRP_8–37_ treatment group, when compared to the saline treated group. (**B**) Total labeled bone area (Tt.L.B.Ar) was also increased in the right ulnae of both treatment groups, and the left ulna of the CGRP_8–37_ treatment group, when compared to the saline treated group. (**C**) Endosteal labeled bone area (Es.L.B.Ar) was increased in the right ulna of both the CGRP and CGRP_8–37_ treatment groups, when compared to the saline treated group. . * −*p*<0.05; ** −*p*<0.01; *** −*p*<0.001 versus the relevant saline control bone. Error bars represent standard error of the mean. Saline group, n = 8; CGRP group n = 12; CGRP_8–37_ group n = 12.

The effect of CGRP and CGRP_8–37_ on load-induced bone formation after cyclic fatigue was evaluated in both the fatigue-loaded right ulnae and contralateral left ulnae. Rats treated with either CGRP or CGRP_8–37_ for 10 days after cyclic fatigue loading had increased reparative bone formation, when compared to the rats that were treated with saline (Fig. 6). Ps.L.B.Ar was increased in the right ulnae of the CGRP (*p*<0.001) and the CGRP_8–37_ (*p*<0.001) groups, and in the left ulna of the CGRP_8–37_ group (*p*<0.0001), when compared to the Saline group; Ps.L.B.Ar was also increased in the left ulna of the CGRP_8–37_ group when compared to the CGRP group (Fig. 7A, *p*<0.05). Similarly, Tt.L.B.Ar was increased in the right ulnae of the CGRP (*p*<0.001) and the CGRP_8–37_ (*p*<0.0001) groups, and in the left ulna of the CGRP_8–37_ group (*p*<0.05), when compared to the Saline group (Fig. 7B). Es.L.B.Ar was also increased in the right ulna of the CGRP (*p*<0.05) and the CGRP_8–37_ (*p*<0.001) groups, when compared to the Saline group (Fig. 7C). No differences in humeral bone formation were seen between any of the groups.

## Discussion

CGRP has been recognized as a neurotransmitter involved in the regulation of bone remodeling and fracture healing [39], [40]. However, the potential effects of CGRP signaling on skeletal responses to bone loading and stress injury have not been investigated. In the present study, using the rat ulna end-loading model to induce cyclic fatigue in a single bone, we determined how CGRP and the CGRP_1_ receptor antagonist, CGRP_8–37_, influenced skeletal repair responses to stress injury, including bone remodeling and load-induced bone new formation. We also determined how cyclic fatigue loading influenced bone CGRP concentrations both locally and at distant skeletal sites. We found that bone CGRP concentrations were increased after unilateral fatigue loading in both loaded and contralateral ulnae. We also found that both CGRP and CGRP_8–37_ augmented new bone formation in response to fatigue but did not influence plasma TRAP5b concentrations. As expected, plasma CGRP concentrations were higher in rats given supplementary CGRP, as compared to rats that were treated with saline or CGRP_8–37_.

TRAP5b is a marker of bone remodeling [41]. Both immature and mature osteoclasts express TRAP5b, and plasma concentrations of TRAP5b are proportional to osteoclast number [42]. Our data shows that systemic administration of CGRP or CGRP_8–37_ did not affect TRAP5b concentrations in plasma, suggesting that 10 days after fatigue loading, overall numbers of activated osteoclasts in each group were not influenced by these treatments. Previous work from our laboratory has suggested that TRAP5b serum concentrations may be neuronally-regulated in fatigue-loaded male rats [43]. The data from this study suggest that neuronal signaling effects on plasma TRAP5b are not associated with CGRP signaling in sensory nerve fibers. It should be noted that TRAP5b levels were measured 10 days after fatigue loading; in future work it would be interesting to investigate markers of bone remodeling throughout a 10 day adaptive period, as the effect of CGRP on osteoclast activation may be more prominent earlier in the experimental period after bone loading.

CGRP-immunoreactive nerve fibers are widely distributed throughout bone [12], [44], [45]. During fracture healing, newly formed CGRP fibers can be found in areas with high bone formation rates [40]. The increase in CGRP concentrations 10 days after cyclic fatigue loading of the right ulna likely reflects altered release of CGRP from sensory nerve endings in loaded bone. Furthermore, the increase in CGRP concentrations in the contralateral ulna 10 days after loading supports the concept that mechanical loading of a single long bone may influence skeletal responses in distant bones through a neuronal mechanism [9]. We hypothesize that the CGRP in the contralateral bone is likely derived from the peptidergic sensory innervation of bone [12]–[15]. It is also possible that bone cells themselves may act as a source of CGRP, although expression in bone cells occurs at a low level [46]. It is well established that cyclic fatigue loading induces a repair response in the rat ulna loading model [37], [47]. Previous work from our laboratory has suggested that loading a single bone may result in a systemic neurovascular response and increased bone formation in distant skeletal long bones, and that these changes are neuronally regulated [9], [43], [47]. CGRP may thus be part of a previously unrecognized neuronal mechanism that regulates skeletal responses to cyclic mechanical loading. In the first experiment in which we studied CGRP concentrations in bone after loading [9], we found decreased bone CGRP concentrations after loading without bone fatigue. Thus, it appears that the release of CGRP into bone tissue is modulated by the intensity and pattern of the applied mechanical signal. However, it is also possible that our results could be influenced by the methodology used to isolate neuropeptides from bone, as we used a different peptide isolation method in the previous study.

Interestingly, we found that systemic administration of CGRP and CGRP_8–37_ over a 10 day period after unilateral fatigue loading increased bone formation in the periosteal, endosteal and intracortical envelopes. The increase in reparative bone formation with CGRP administration was anticipated; CGRP has been shown to be an inhibitor of bone resorption [48], and increases osteogenesis in a dose-dependent manner [49].

CGRP_8–37_ principally antagonizes signaling via the CGRP_1_ receptor [20], [23], [24]. This receptor is well defined as a heterodimer of the calcitonin receptor-like receptor (CRLR) and receptor activity modifying protein 1 (RAMP_1_) [23], [50]. In addition, CGRP is also thought to signal through a putative CGRP_2_ receptor [23]. In rodents, CGRP binds to variants of both adrenomedullin (AM) (AM_2_ consisting of CRLR and RAMP_3_) and amylin (AMY) receptors (AMY_1(a)_, AMY_3(a)_) [23], [51], [52]. AMY_1(a)_ consists of RAMP_1_ and an insert negative form of the calcitonin receptor (CTR), while AMY_3(a)_ consists of RAMP_3_ and the same CTR variant [23], [52]. Thus the ‘CGRP_2_ receptor’ may in reality be an amalgamation of contributions from a variety of CGRP-activated receptors [23].

As CGRP has been shown to activate bone formation, it is not surprising that CGRP-α knockout mice are osteopenic [32]. The increase in bone formation in rats treated with CGRP_8–37_, however, was an unexpected result. The heterogeneity of CGRP receptor signaling may explain this observation, and suggests that signaling via a CGRP receptor that is not CGRP_1_ is responsible for the load-induced osteogenic response. However, CGRP also has actions on multiple organ systems, and it is also possible that the effect of CGRP on vascular tone may help explain this result [53]–[58]. Increased bone blood flow precedes bone repair in response to fatigue loading, and remodeling in response to decreased mechanical loading [47], [58]. Systemic administration of CGRP also decreases blood pressure in a dose-dependent manner [59]. Therefore, treatment with CGRP_8–37_ may have increased intraosseus pressure, transcortical interstitial fluid flow, and associated bone formation [60], [61], although such an effect appears less likely in our model since we did not detect significant changes in humeral bone formation with CGRP or CGRP_8–37_ treatment.

As expected, 10 days after cyclic fatigue loading there were no differences in Cr.S.Dn between groups, indicating that rats in each group experienced a similar degree of fatigue damage during bone loading. However, we found that treatment with CGRP_8–37_ did have a significant effect on osteoclast recruitment and activation for targeted remodeling in the fatigue-loaded ulna; targeted remodeling in the fatigue-loaded ulna was increased after treatment with CGRP_8–37_. CGRP-immunoreactive nerve fibers have direct contact with osteoclasts [18]. CGRP is an inhibitor of bone resorption, possibly through interference with the action of the receptor activator of NF-kB (RANKL), and thus differentiation and recruitment of osteoclast precursors [48], [62]. Our results suggest the CGRP_1_ receptor is responsible for this in-vivo effect, such that in fatigue-loaded rats treated with the CGRP_8–37_, the normal inhibition of CGRP on osteoclast activation for targeted remodeling appears diminished. Interestingly, this effect was detected in the face of increased bone CGRP concentrations in both loaded and contralateral ulnae at 10 days and a lack of difference in plasma TRAP5b between groups, suggesting that the signals regulating targeted remodeling of damaged bone are complex, and may involve other pathways that activate osteoclastic bone remodeling. CGRP signaling may have greater effects on osteoclastic activation, and a lesser effect on osteoclastic recruitment and proliferation *in vivo*. We also detected a small CGRP treatment effect on remodeling in the contralateral ulna; the biological significance of this observation is unclear.

A limitation to this study is that we analyzed bone CGRP concentrations and bone repair at a single time point, 10 days after fatigue loading. Additionally, it is not possible to fully isolate direct effects of CGRP signaling on bone cells from effects of CGRP on neuronal signaling, and possibly bone blood flow. As both CGRP and CGRP_8–37_ augmented reparative bone formation, our data suggest that CGRP release from nerve endings during bone loading acts to modulate load-induced skeletal repair. Our results implicate the CGRP_1_ receptor as responsible for effects on osteoclasts, and a CGRP receptor that is not CGRP_1_ as responsible for the osteogenic effects we observed.

In conclusion, our data support the established hypothesis that CGRP is osteoanabolic to the skeleton in that it acts to increase osteogenesis and inhibit osteoclastic bone resorption *in vivo* in a bone fatigue model. The present study also suggests that CGRP has regulatory effects on skeletal responses to mechanical loading. Our results suggest that CGRP augments load-induced bone formation and inhibits osteoclastic remodeling through increased release of CGRP from the peptidergic sensory innervation of bone tissue that is being repaired. Future work should confirm whether or not mechanical loading of the skeleton leads to up-regulation of CGRP in the sensory innervation of loaded bone, as is thought to occur during nociception [63], and also determine which CGRP receptors are responsible for these actions.
